# Necrological

**Published:** 1881-01

**Authors:** 


### NECROLOGICAL.

" Death is the crown of life:
Were death denied, poor man would live in vain."

WILLIAM NEWTON, infant son of Dr. Harvey L. and Mrs. Florence Byrd, was called to his home among the angels, December 14, 1880.

We learned of the death of Dr. Benjamin W. Hardee, of Savannah, Georgia, a few weeks ago. Dr. Hardee was a man of warm and generous impulses, and integrity of character. He began and continued his medical studies in our office, and under our supervision up to the time of his graduation as a Physician, a quarter of a century ago, and we recall many pleasing reminiscences of those days. For many years our fields of labor have been widely separated, but we have a good report of his quiet, but useful career. Peace to his ashes.

